# The Effect of Bilateral Nasal Sphenopalatine Ganglion Block in Managing Headaches After Dural Puncture Following Lower Segment Cesarean Section: A Prospective Observational Study

**DOI:** 10.7759/cureus.80289

**Published:** 2025-03-09

**Authors:** Pushpraj Singh, Tanmoy Ghatak, Utsav Anand Mani, Alka Verma, Asif Dabeer Jafri, Shyam Sundar, Melissa Lourdes Carlos, Malika Dhawal

**Affiliations:** 1 Anesthesiology, Career Institute of Medical Sciences and Hospital, Lucknow, IND; 2 Emergency Medicine, Sanjay Gandhi Postgraduate Institute of Medical Sciences, Lucknow, IND; 3 Internal Medicine, SUM Ultimate Medicare, Bhubaneswar, IND; 4 Anesthesiology, P. D. Hinduja Hospital & Medical Research Centre, Mumbai, IND; 5 Anesthesiology, King George's Medical University, Lucknow, IND

**Keywords:** epidural blood patch (ebp), lower segment cesarean section (lscs), post-dural puncture headache (pdph), sphenopalatine ganglion block (spgb), spinal and epidural anesthesia

## Abstract

Background: Post-dural puncture headache (PDPH) is a common complication that occurs in a small percentage of patients who undergo spinal or epidural anesthesia. The current treatment modalities for PDPH involve both conservative management and interventional approaches. In cases where conservative management is ineffective or if the symptoms are severe, interventional treatments are considered. The gold standard treatment for PDPH is the epidural blood patch (EBP). In recent years, minimally invasive interventions such as sphenopalatine ganglion block (SPGB) have been practiced, where a local anesthetic is injected into the sphenopalatine ganglion to block pain signals.

Objective: The primary objective of this study was to study the effectiveness of bilateral intranasal SPGB for the management of PDPH after lower segment cesarean section (LSCS).

Methods: Hundred parturients diagnosed to have PDPH were recruited into this prospective observational study. Patients were allocated to either of the two groups. Descriptive statistics, the chi-squared test, and Student's t-test were used for statistical analysis.

Results: The onset of analgesia in the SPGB group was 15 times faster than that of the conservatively treated control group. There was a reappearance of PDPH with a visual analogue scale(VAS) score >4 in three instances after SPGB.

Conclusion: SPGB is a very effective initial modality for managing severe headaches in patients with PDPH. Our study, beyond a reasonable doubt, indicates the excellent efficacy of SPGB over conservative management alone.

## Introduction

The International Headache Society (IHS) characterizes post-dural puncture headache (PDPH) as a type of headache that arises within five days following a lumbar puncture, resulting from the leakage of the cerebrospinal fluid (CSF) through the dural puncture site. PDPH is a common and distressing complication characterized by severe postural headache and may be associated with nausea and vomiting, neck and back pain, tinnitus, and, rarely, diplopia [1-3]. Typically, epidural analgesia is frequently employed to manage labor pain, with usage rates exceeding 50% in numerous healthcare facilities across the United States and surpassing 85% in specialized labor and delivery centers that provide round-the-clock obstetric anesthesia services. However, the administration of labor epidural analgesia is not devoid of risks; the most prevalent complication associated with the placement of an epidural catheter is unintentional dural puncture (UDP), which occurs in approximately 0.51-1.5% of obstetric patients. Furthermore, it is estimated that 50-80% of these patients may experience a PDPH. This headache begins within 48 hours after an epidural needle placement and, if not treated, tends to resolve on its own within approximately two weeks for most women, although it may persist longer for some individuals. Various factors that affect the occurrence of PDPH include age, sex, prior headache history, characteristics of the needle used, the number of puncture attempts, and the clinical expertise of the healthcare provider. One of the most common complications of spinal anesthesia or dural puncture is PDPH. Dural puncture can lead to the excessive leakage of CSF, which can, in turn, lead to intracranial hypotension and reduction in CSF volume, which is the cause of the headache. The incidence of PDPH is high in the obstetric population because of their young age, sex, and widespread use of central neuroaxial blocks [4]. Epidural blood patch (EBP) is the treatment of choice in these cases, and therapeutic evidence-based practice demonstrates success rates varying from 68% to 90%; however, it is accompanied by potential complications such as subdural hematoma, infection, meningitis, and delayed radicular pain [5,6]. So, here, we conducted the study that the administration of sphenopalatine ganglion block (SPGB) in patients with PDPH will reduce the severity of pain and it may be a useful modality in PDPH patients who do not get relief by conservative management with oral analgesics and caffeine.

## Materials and methods

This prospective observational study was carried out at the Department of Anesthesiology at Baba Raghav Das Medical College, Gorakhpur, Uttar Pradesh, India. The research received ethical approval from the institute's Institutional Ethics Committee (approval number: 412/Ethics/2019), and written informed consent was obtained from all participating patients. The study was focused on adult female patients, aged 18-45 years and classified as American Society of Anesthesiologists (ASA) grades I-II, who underwent (elective or emergency) cesarean section under spinal anesthesia with 25-gauge Quincke spinal needle followed by suggestive features of PDPH. This research was conducted from December 2018 to November 2019 at Baba Raghav Das Medical College, where approximately 20 patients undergo lower segment cesarean sections (LSCS) daily, totaling an average of 5000-6000 LSCS annually. We included a total of 100 female patients, 50 in two groups (C group for conservative and S group for SPGB): the C group received conservative treatment (bed rest, fluid therapy, tablet paracetamol 500 mg three times a day (TDS) orally, tablet caffeine 100 mg one tablet TDS orally, and tablet diclofenac 75 mg twice daily (BD) orally) and the S group received bilateral SPGB via intranasal route with local anesthetic 1-2 ml, 4% lignocaine solution.

The intranasal route for SPGB was chosen. Then the block was achieved by inserting a long cotton-tipped applicator soaked in local anesthetic with 1-2 ml, 4% lignocaine solution. During the initial hours of assessment, if the visual analogue scale (VAS) score was >4, then conservative management is added, and the block was to be repeated if the VAS score was >4 after 24 hours. After the aforementioned management, if the patient still complained of a headache, then the patient was to be shifted to another modality of treatment which was EBP (which would then be excluded from this study). VAS score for headache was recorded before initiating treatment, 30 minutes after initiating treatment, at four, 12, 24, 48, and 72 hours, and at discharge from the hospital.

The inclusion criteria for this study consist of patients who are classified as ASA I and II, those who have confirmed cases of PDPH occurring within seven days after a subarachnoid block (SAB), post-operative cesarean patients between the ages of 18 and 45 years, patients who underwent LSCS (both emergency and elective), and those who have received SAB. The exclusion criteria include patients who refuse participation, those with a local infection at the injection site, and individuals with spinal deformities, bleeding disorders, or allergies to local anesthetics. Also excluded are patients with contraindications to the proposed anesthetic technique, ASA ≥III patients, individuals with a history of chronic pain or daily use of central nervous system (CNS) medications, as well as those with a history of other headache types such as non-specific post-natal headache, migraine, pre-eclampsia, meningitis (septic or aseptic), intracranial hemorrhage, mass lesions, cerebral vein thrombosis, post-natal depression headache, or pneumocephalus. Additionally, patients with nasal septal deviation, polyps, or a history of nasal bleeding are excluded from the study.

Statistical analysis

All statistical analyses were conducted using IBM SPSS Statistics for Windows, V. 26.0 (IBM Corp., Armonk, NY, USA). Descriptive statistics were utilized to summarize the demographic characteristics and clinical parameters of the study participants. Continuous variables, such as VAS scores at different time intervals and the onset time of analgesia, were presented as mean±standard deviation (SD). Student's t-test (independent samples t-test) was employed to compare the mean VAS scores between the two groups at each time point. In cases where the normality assumptions were violated, as determined by the Shapiro-Wilk test, the Mann-Whitney U test was applied as an alternative for comparing continuous variables across groups. Categorical variables, such as the occurrence of PDPH recurrence and the need for additional interventions, were reported as counts (n) and percentages (%) and analyzed using either the chi-squared test or Fisher's exact test, based on whether any expected frequencies in the cells were below five. For the analysis of repeated measures, since VAS scores were collected at multiple time points within each group, a repeated measures ANOVA or Friedman test (for non-parametric data) was employed to assess changes over time within each group. A p-value of less than 0.05 was deemed statistically significant for all analyses.

## Results

**Figure 1:**
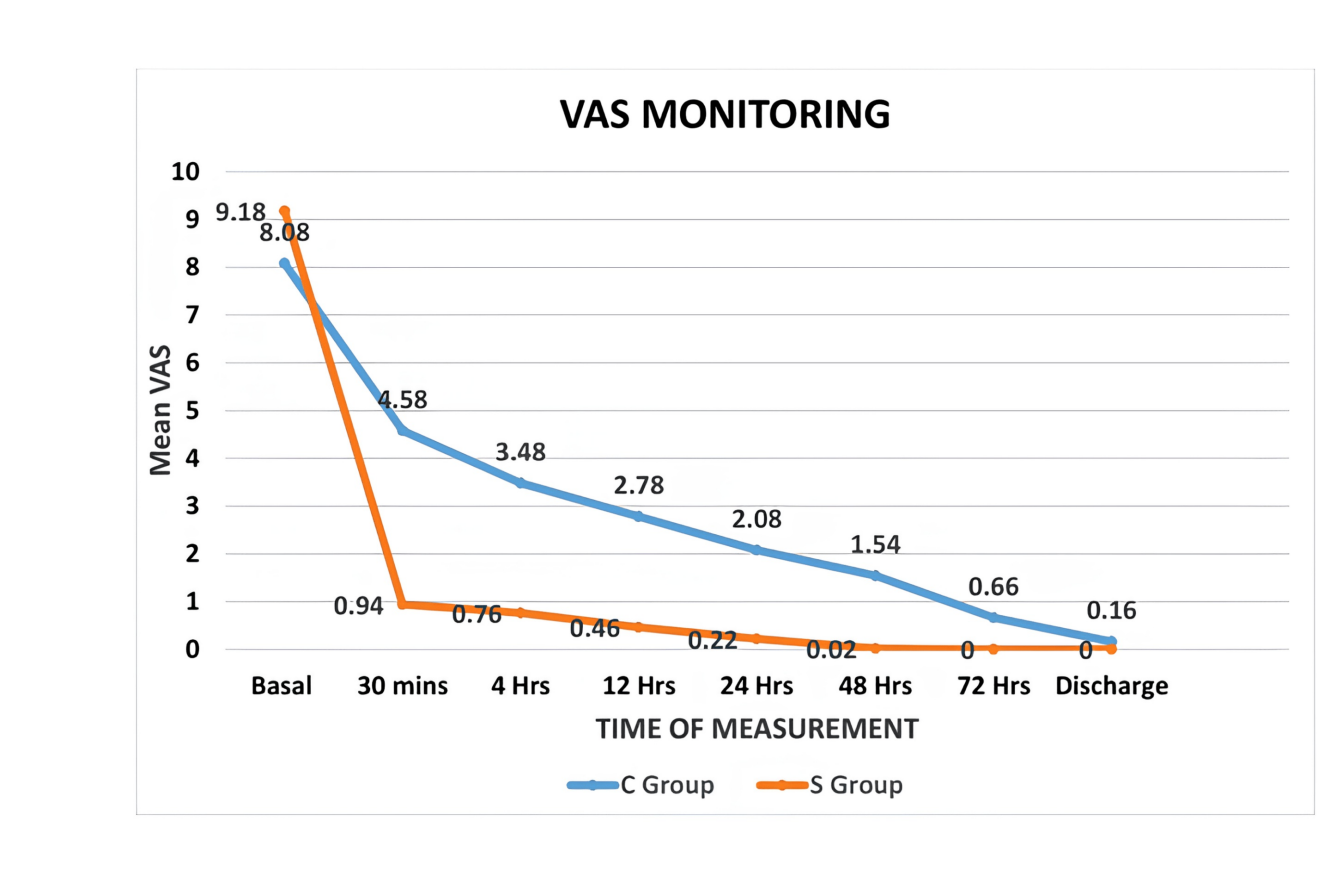
VAS monitoring VAS: visual analogue scale

Comparison of basal VAS score revealed that the mean VAS for the S group (9.18±1.21) was higher than that of the C group (8.08±1.20) which was statistically very significant (p<0.00001). After giving block in the S group and conservative management in the C group, the VAS decreased drastically in both groups. The analysis between the groups revealed the following: the mean VAS at 30 minutes for the S group was 0.94±1.12 and for that of the C group was 4.58±0.70, which was statistically very significant (p<0.00001). The mean VAS at four hours for the S group was 0.76±1.19 and for that of the C group was 3.48±0.75, which was statistically very significant (p<0.00001). Similarly, at 12 hours, VAS was 0.46±1.17 (S group) and 2.78±0.76 (C group), which was statistically very significant (p<0.00001). At 24 hours, it was 0.22±0.70 in the S group and 2.08±0.66 in the C group, which was statistically very significant (p<0.00001). At 48 hours, VAS was 0.02±0.14 in the S group and 1.54±0.81 in the C group, which was statistically very significant (p<0.00001). At 72 hours, VAS was 0.00±0.00 and 0.66±0.76 for the S and C groups, respectively, which was statistically significant (p<0.00001). Finally at discharge, VAS was 0.00±0.00 and 0.16±0.37 for the S and C groups, respectively, which was statistically significant (p=0.0036). The results of the study are shown in Table 1 and Figure 1.

**Table 1:** VAS monitoring p<0.05: statistically significant. VAS: visual analogue scale

Time in minutes	C group (50)		S group (50)		Test statistics	P-values
	Mean	SD	Mean	SD		
Basal	8.08	1.20	9.18	1.21	-5.001	<0.00001
30 minutes	4.58	0.70	0.94	1.12	21.304	<0.00001
4 hours	3.48	0.75	0.76	1.19	13.325	<0.00001
12 hours	2.78	0.76	0.46	1.17	11.107	<0.00001
24 hours	2.08	0.66	0.22	0.70	13.28	<0.00001
48 hours	1.54	0.81	0.02	0.14	13.201	<0.00001
72 hours	0.66	0.76	0.00	0.00	6.0434	<0.00001
Discharge	0.16	0.37	0.00	0007	3.0550	0.0036

The mean duration for the onset of analgesia, measured in minutes, was assessed for the two groups: the C group and the S group. The C group exhibited a mean onset time of 180.60 minutes, with an SD of 33.97. In contrast, the S group demonstrated a considerably shorter mean onset time of 11.6 minutes, with an SD of 2.37. The results of the statistical analysis revealed a t-value of 34.365 and a p-value of less than 0.00001, signifying a highly significant difference between the two groups (p<0.05). The onset of analgesia in the S group was 15.57 times faster than that of the conservatively treated C group.

The quickest time for the onset of analgesia in the S group was 10 minutes, and the longest time was 20 minutes. In the C group, it was 120 minutes and 240 minutes, respectively.

## Discussion

To perform SPGB, the patient must be positioned supine with the neck extended. A long applicator, equipped with a cotton swab at its tip and saturated with a local anesthetic, is inserted parallel to the nasal floor until resistance is felt. At this juncture, the swab will be positioned at the posterior pharyngeal wall, just above the middle turbinate. The applicator should remain in place for five minutes before being withdrawn. This procedure must then be repeated in the opposite nostril. Potential complications associated with the transnasal approach may include mild discomfort during the procedure, bleeding, and throat numbness [7].

The SPGB is characterized by its minimally invasive nature, resulting in few side effects while providing effective and rapid pain relief. When employed as the primary treatment for PDPH, it offers quicker analgesia compared to conservative methods. Utilizing this technique can eliminate the need for an EBP, which is an invasive procedure that carries potential complications. SPGB can be administered through various approaches, including transnasal, transoral, sub-zygomatic, and lateral infratemporal methods. Among these, the transnasal approach is the simplest and least invasive, making it suitable for bedside application. Consequently, we chose this method for our study. The effectiveness of SPGB in alleviating pain associated with PDPH has been well established, and it is regarded as a safe procedure, with contraindications limited to local nasal infections and fractures at the base of the skull [8-10].

In alignment with our findings, Puthenveettil et al. determined that the use of SPGB resulted in more rapid and effective relief compared to conservative treatment [11]. Their study indicates that SPGB may serve as an effective initial approach for the management of PDPH, facilitating swift alleviation of severe pain, which is consistent with the results of our research. Additionally, our study observed a consistent reduction in pain scores leading up to discharge. Cohen et al. conducted a retrospective study comparing SPGB and EBP in 81 patients [12]. At the end of one hour, SPGB patients had good pain relief compared to EBP patients; after 24 hours, no significant difference was observed. Moreover, more complications were observed in the EBP group.

In our study, the patients had steady pain relief in the block group till discharge. None of them required a repeat block. However, three patients required conservative therapy post-block. As reported by Vallejo et al. and Kent and Mehaffey, adequate pain relief was obtained with 2% lignocaine as well as ropivacaine when used to perform SPGB in obstetric patients with PDPH [13,14]. These patients had pain relief for 12-24 hours. In our study, 4% lignocaine was used in all patients which provided early onset and prolonged effect of pain relief.

Nair and Rayani, in their narrative review on efficacy, concluded that the SPGB is a straightforward and minimally invasive procedure that can be performed at the bedside. Upon examining the existing evidence, the reviewers recommended, "We can offer the SPGB to all patients diagnosed with moderate to severe PDPH. If the block does not effectively relieve pain, an EBP can be considered [8]. Clinicians should maintain ongoing supportive management following the administration of the SPGB." Our study aligns with this perspective, suggesting that a combined regimen may be more practical.

Cardoso and colleagues concluded that the SPGB may reduce cerebral vasodilation caused by parasympathetic stimulation relayed through neurons with synapses in the sphenopalatine ganglion [15]. This finding aligns with the Monro-Kellie doctrine and provides insight into the efficacy of caffeine and sumatriptan in managing PDPH. In our research, we administered non-steroidal anti-inflammatory drugs (NSAIDs), caffeine, bed rest, and adequate hydration within the conservative treatment group. Additionally, the authors noted that SPGB appears to have a quicker onset compared to EBP and a more favorable safety profile. The researchers recommended that patients experiencing PDPH should primarily be considered for SPGB, with EBP available as a secondary option if necessary.

We observed minor complications like bilateral nasal congestion in one patient and dizziness and throat congestion in two patients post-block; however, during treatment or post-intervention, no other adverse events were observed in both groups. There is no evidence of serious complications related to SPGB in our study. There have been no specific reported complications of bilateral SPGB PDPH with a VAS score >4 in three instances after SPGB.

## Conclusions

SPGB is recognized as a highly effective initial intervention for the relief of severe headaches in patients suffering from PDPH. Our findings clearly indicate that the SPGB outperforms conservative management alone in terms of efficacy. The SPGB is acknowledged as a viable treatment for PDPH, demonstrating a favorable success rate when compared to conservative therapies. Nevertheless, before it can be routinely implemented in clinical practice, well-structured randomized controlled trials (RCTs) are necessary to confirm and validate its effectiveness.
